# Dosimetric comparison and evaluation of two computational algorithms in VMAT treatment plans

**DOI:** 10.1002/acm2.14051

**Published:** 2023-06-21

**Authors:** Maria Tsimpoukelli, George Patatoukas, Marina Chalkia, Nikolaos Kollaros, Andromachi Kougioumtzopoulou, Dimitra Michaletou, Vassilis Kouloulias, Kalliopi Platoni

**Affiliations:** ^1^ 2nd Department of Radiology, Medical Physics Unit, School of Medicine, Attikon University Hospital National and Kapodistrian University of Athens Athens Greece; ^2^ 2nd Department of Radiology, Radiation Oncology Unit, School of Medicine, Attikon University Hospital National and Kapodistrian University of Athens Athens Greece

**Keywords:** AAA, Acuros XB, dose calculation, volumetric modulated arc therapy

## Abstract

**Purpose:**

This study aimed to assess the accuracy and dosimetric impact of the Acuros XB (AXB) algorithm compared to the Anisotropic Analytical Algorithm (AAA) in two situations. First, simple phantom geometries were set and analyzed; moreover, volumetric modulated arc therapy (VMAT) clinical plans for Head & Neck and lung cases were calculated and compared.

**Methods:**

First, a phantom study was performed to compare the algorithms with radiochromic EBT3 film doses using one PMMA slab phantom and two others containing foam or air gap. Subsequently, a clinical study was conducted, including 20 Head & Neck and 15 lung cases irradiated with the VMAT technique. The treatment plans calculated by AXB and AAA were evaluated in terms of planning target volume (PTV) coverage (V_95%_), dose received by relevant organs at risk (OARs), and the impact of using AXB with a grid size of 1 mm. Finally, patient‐specific quality assurance (PSQA) was performed and compared for 17 treatment plans.

**Results:**

Phantom dose calculations showed a better agreement of AXB with the film measurements. In the clinical study, AXB plans exhibited lower Conformity Index and PTV V_95%_, higher maximum PTV dose, and lower mean and minimum PTV doses for all anatomical sites. The most notable differences were detected in regions of intense heterogeneity. AXB predicted lower doses for the OARs, while the calculation time with a grid size of 1 mm was remarkably higher. Regarding PSQA, although AAA was found to exhibit slightly higher gamma passing rates, the difference did not affect the AXB treatment plan quality.

**Conclusions:**

AXB demonstrated higher accuracy than AAA in dose calculations of both phantom and clinical conditions, specifically in interface regions, making it suitable for sites with large heterogeneities. Hence, such dosimetric differences between the two algorithms should always be considered in clinical practice.

## 1 INTRODUCTION

The effectiveness of radiation therapy depends on the ability to maximize the tumor coverage while minimizing the dose received by adjacent normal tissues. This can be achieved by delivering an appropriate therapeutic radiation dose calculated and directed with maximum accuracy to the planning target volume (PTV). Therefore, accurate knowledge of dose distribution within the patient's body is vital and requires elaborate calculations by the dose calculation algorithm of the treatment planning system (TPS).

During the last decades, modern radiotherapy techniques are constantly being evolved and applied, such as intensity‐modulated radiotherapy (IMRT), volumetric modulated arc therapy (VMAT), and stereotactic body radiation therapy (SBRT). These advancements, in conjunction with the application of hypofractionated radiotherapy dose prescription schemes, make the need for accurate dose calculation even greater. International Commission on Radiation Units and Measurements (ICRU) has recommended an accuracy of 5% in the delivery of absorbed dose to a target volume.^1^ Considering all other sources of uncertainty, such as machine calibration, patient setup, and dose calculation from TPS, the dose calculation algorithm should predict dose distribution within 3% accuracy.^2^

Accurate calculation of dose distribution in the human body can demand complicated calculations in heterogeneous regions, particularly in head & neck and lung tumors. The transient absence of electron equilibrium and the increased lateral range of electrons in the air result in a significant reduction of central axis dose beyond the cavity and potentially a tumor underdosage.^3^ In order to correct for the heterogeneity and to obtain an accurate dose calculation, the calculation algorithm used by the TPS should take into account the lateral scattering of electrons.

The Anisotropic Analytical Algorithm (AAA) is a model‐based algorithm that uses convolution/superposition techniques to calculate the dose, considering the contribution of scattered photons and electrons described by a kernel.^4^ The dose is calculated by the convolution of the total energy released per unit mass (TERMA) with this kernel. The heterogeneity correction is performed only in the longitudinal and lateral direction (±x and ±y) by applying a radiological density scaling to account for the different local electron density compared to water (superposition). The accuracy of dose calculation on heterogeneous media depends on how well the kernel of the algorithm can simulate the actual scattering.^5^ AAA provides improved accuracy compared to previous algorithms and is widely used in radiotherapy TPS as it provides high accuracy and short calculation times. However, since the calculations take into account only the density difference of the tissues and not their elemental properties, there is a limit to the dose accuracy in regions with extreme changes in biological tissues density (e.g., lung, air, bone) and high atomic number‐ (Z) implanted materials.^6^

Acuros XB (AXB) is a new generation algorithm based on the explicit solution of the linear Boltzmann transport equation (LBTE) and provides calculation accuracy comparable to full Monte Carlo methods but with a faster calculation time.^7^ It uses the same multiple‐source model as AAA, but it calculates the treatment plan dose in two phases. In the first phase, it simulates the propagation of the radiation beam in the linear acceleration head, while in the second phase it calculates the dose distribution in the patient body. AXB takes into account the elemental properties of each anatomical region tissue, matching each geometry voxel with a mass density and a material type based on the patient's CT.^8^ Thus, it achieves high accuracy calculations, even in heterogeneous regions with high‐density contrast where other algorithms produce significant discrepancies. The AXB algorithm inherently calculates dose‐to‐medium (Dm,m). However, the dose distributions can be converted to dose‐to‐water (Dw,m) by replacing the medium‐based fluence‐to‐dose response function used in absorbed dose calculation with a water‐based response function.^8^ However, as this conversion involves some uncertainty, Dm,m might be preferable for treatment plan evaluation and outcome analysis.^9^, ^10^ Nevertheless, the question of which dose reporting mode should be used in clinics has not yet been clearly answered, and further investigation is needed.^11^, ^12^

The accuracy of the AXB algorithm compared to the AAA has been investigated in various studies. Kroon et al. studied the accuracy of the two algorithms in a heterogeneous phantom and found that AXB best predicts the actual dose distribution in cases of very low‐density heterogeneity, where AAA overestimates the dose.^13^ When assessing VMAT therapy plans for lung cancer, they concluded that AXB is preferable to AAA to avoid serious PTV underdosage (D_98%_ is predicted higher by AAA). Also, Padmanaban et al. found that in esophageal treatment plans created with the RapidArc technique, AXB predicted a significantly lower average dose in PTV, Gross Tumor Volume (GTV), and Organs At Risk (OARs), with dose differences being higher in areas where soft tissue is surrounded by low‐density lung.^14^ They concluded that AAA overestimates the dose in PTV and results in its underdosage, which could lead to a different tumor control probability. Additional studies concerning prostate and head & neck (H&N) cases end up with similar conclusions.^15^, ^16^, ^17^

Even though the dosimetric behavior of the AXB algorithm has been examined in several studies, we feel that conclusions regarding the use and properties of the AXB appear to be sporadic, and thus a thorough, detailed, and exhaustive study in both phantom and clinical scenarios can add and contribute to a deeper understanding of this dose calculation algorithm's impact on the various aspects of treatment planning. Thus, the purpose of this study was twofold. First to evaluate the accuracy of the AXB algorithm in homogeneous and heterogeneous conditions, and second, to assess the dosimetric impact of the AXB in usual clinical scenarios. For the latter, we compared the two algorithms in VMAT plans for two different anatomical sites in terms of dosimetric result, calculation time, and their impact on the quality of the deliverability of the treatment plan.

## 2 MATERIALS AND METHODS

Firstly, a phantom study was conducted in which doses measured by radiochromic films were compared to the calculated ones by the TPS using the AXB and AAA algorithms. The measurements were performed in a homogeneous polymethyl methacrylate (PMMA) slab phantom and in two heterogeneous phantoms: one containing a low‐density foam block and another containing an air gap between PMMA slabs. Measurements at several depths and interface positions between the different materials were taken into consideration. Secondly, VMAT treatment plans for lung and H&N cases were calculated with both algorithms to evaluate their dosimetric differences and compare them with the phantom study results. The AXB plans were not re‐optimized, and the number of MUs was kept the same in order to compare the AXB calculated dose to the AAA one. A grid size of 2.5 mm was used for the dose calculation and additionally, for reasons of comparison, a 1 mm grid size was also considered.

### 2.1 Phantom study

**FIGURE 1:**
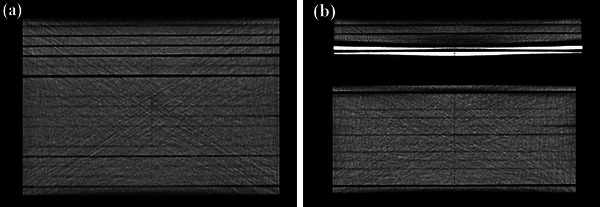
CT image of homogeneous PMMA slab phantom (a) and heterogeneous PMMA slab phantom with foam or air gap (b).

**FIGURE 2:**
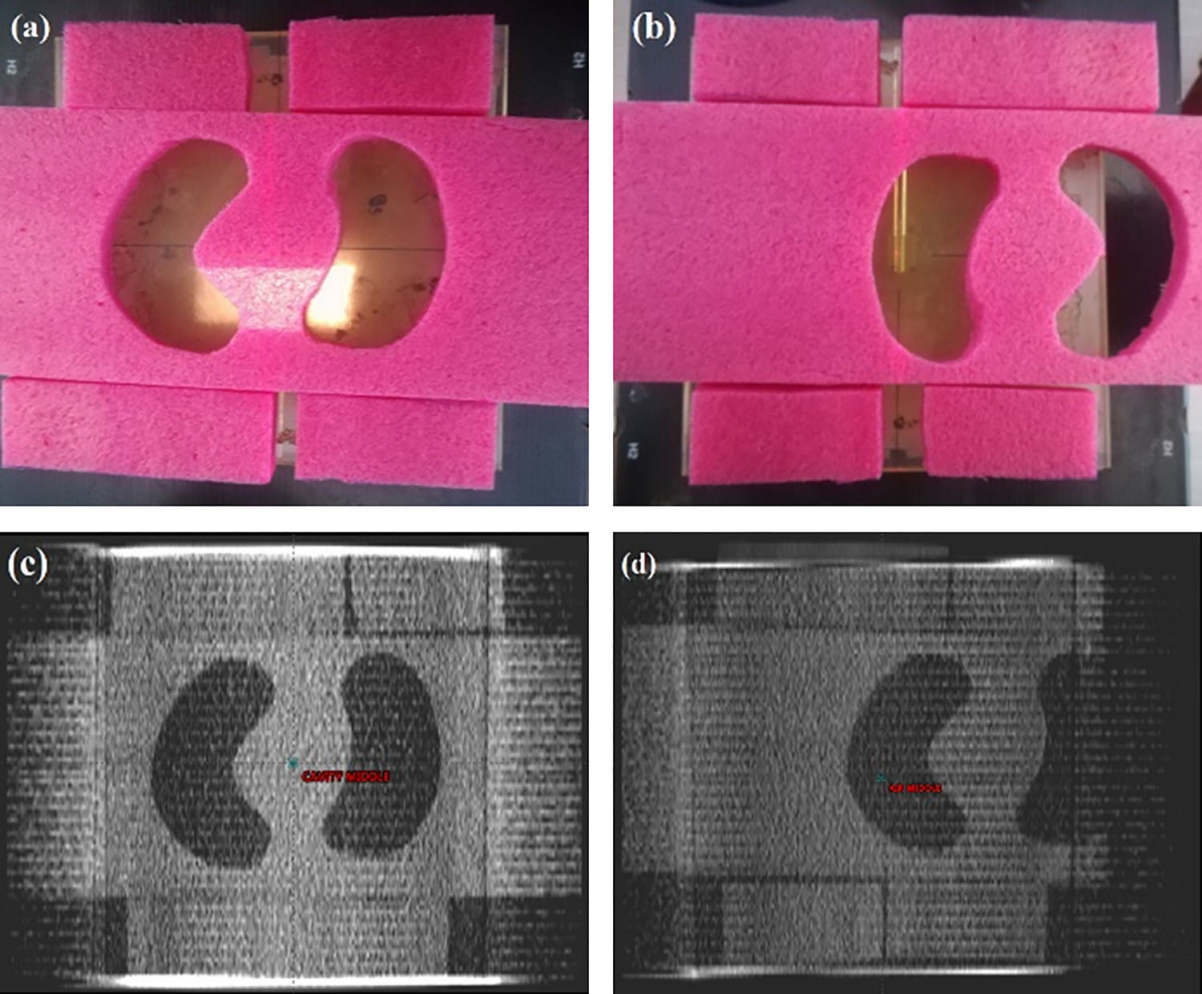
(a) The heterogeneous phantom with foam heterogeneity above the film, (b) the heterogeneous phantom with air heterogeneity above the film, (c) and (d) the corresponding CT transverse sections of heterogeneous phantoms at the depth of heterogeneity.

Three different phantoms were constructed, depicted in Figures 1 and 2. The first one was a homogeneous phantom consisting of PMMA slabs (30×30 cm^2^) of a total height of 18 cm (Figure 1a). The second one was a heterogeneous PMMA slab phantom containing a 3 cm low‐density foam block between 18 cm (beyond) and 5 cm (above) PMMA slabs (Figure 1b). The third one was a heterogeneous phantom containing a 3 cm air cavity between 18 and 5 cm PMMA slabs. The heterogeneity was introduced in the phantom simply by displacing the different foam parts so that the film would lie beyond the air or foam heterogeneity (Figure 2).

For each phantom, irradiations with three photon energies of 6 MV, 10 MV, and 6 MV FFF (flattering filter‐free) were performed on a Varian VitalBeam™ linear accelerator (Varian Medical Systems, Palo Alto, CA). At each irradiation, 100 monitor units (MUs) were delivered with an open field size of 10×10 cm^2^, and a source‐to‐skin distance (SSD) of 100 cm. For the dose measurements, EBT3 radiochromic films were used and handled following the guidelines of AAPM Task Group 235.^18^ Small rectangular (2 × 2.5 cm^2^) film strips from the same batch were cut and marked for orientation in order to maintain their orientation consistent throughout the measurement process. The films were reproducibly scanned using an EPSON Expression Perfection V850 Pro scanner, which is a flatbed scanner with red, green, and blue color channel capability. Films were placed in the central area of the scanner and evaluated with the corresponding software 72 ± 2 h after irradiation to ensure the stabilization of the polymerized film optical density (OD). The measured doses were point doses in the central area of each film, placed along the central axis of the phantoms.

In the homogeneous phantom, the films were placed at depths of 4 cm, 5 cm, 6 cm, 8 cm, and 10 cm, while in the heterogeneous phantoms, they were placed at depths of 2 and 1 cm above the heterogeneity and 1 cm, 3 cm, 5 cm, and 6 cm below the heterogeneity. For measurements at the interfaces, films were placed in the foam‐PMMA and air‐PMMA interface (8 cm depth from the phantom surface).

Preceding the usage of the films, a film calibration was performed. 2 × 2.5 cm^2^ film strips from the same film batch were irradiated with a 6 MV photon beam at different dose values in a range of 10 cGy‐300 cGy, under the same general conditions as those used in the IAEA TRS‐398 protocol.^19^ The dose to the films was also calculated according to TRS‐398.^19^ The films were scanned 72 ± 2 h after irradiation using the aforementioned scanner. Before scanning the films, 10 warm‐up scans were performed to obtain light source stability. Net OD values were calculated by subtracting the OD values of nonirradiated background films (from the same batch) from the OD values of the calibrated films. The calibration curve was generated by fitting the netOD values to the corresponding film doses. For the dose conversion, empirical fitting functions were used due to the nonlinear calibration curve.

A computed tomography (CT) scan of each phantom was performed with a 3 mm slice thickness, 130 mAs, and 120 kV. All phantoms were aligned using external radiopaque markers (ball bearings ‐BBs) placed on the phantoms before CT simulation. Subsequently, the CT datasets were imported into Varian Eclipse TPS, and treatment plans for both algorithms were created with identical parameters to those used in irradiations. The calculated doses at the same points where the films were placed were recorded.

The dose difference between the algorithm and film was calculated using the (Equation 1):

(1) $$\Delta \left(\%\right)=\left(\frac{D_{AXB\;or\;AAA}-D_{film}}{D_{film}}\right)\;\;\times 100\;$$

Likewise, the differences between the calculated doses with the two algorithms at the same points were calculated using (Equation 2):

(2) $$\Delta \left(\%\right)=\left(\frac{D_{\mathrm{AXB}}-D_{\mathrm{AAA}}}{D_{\mathrm{AAA}}}\right)\times 100$$

### 2.2 Clinical study

#### 2.2.1 Patient selection and dose calculation

For the "clinical" part of the study, twenty H&N and fifteen lung cancer patients treated with VMAT treatment plans were selected from the database of our clinic. As the study was retrospective, the target and OAR delineation had already been performed by radiation oncologists, and the treatment plans had already been optimized and calculated using the AAA algorithm (version 15.1.51) with a 6 MV photon beam and calculation grid size of 2.5 mm. Varian TPS ('Eclipse™v.15.1, Varian Medical Systems) was used to create and calculate the treatment plans. No plan was re‐optimized for reasons of consistency. For each plan, the final dose calculation step was recalculated using the AXB algorithm (version 15.1.51), selecting the dose‐to‐medium calculation mode. The (AAA‐based) optimization resulting fluences were kept the same in order to compare the Acuros calculated dose to the AAA calculated dose. The number of MUs resulting from the optimization, which included the intermediate dose calculation step, was also the same for both algorithms.

#### 2.2.2 Dosimetric plan evaluation

Dose‐volume histograms (DVHs) were generated for the PTV and OARs of each plan to compare the dosimetric characteristics of the plans calculated with the two algorithms. The dosimetric parameters compared for PTV and OARs of each site are summarized in Table 1. For H&N cases, the radiotherapeutic fractionation scheme included three consecutive irradiation phases with different dose prescriptions (sequential boost(s)). PTV1 (50‐54 Gy) included the primary tumor, high and intermediate risk elective nodal regions, and low‐risk lymph nodes (unfiltered, contralateral); PTV2 (60 Gy) included the primary tumor and high and intermediate risk elective nodal regions; and PTV3 (70 Gy) included the primary tumor and positive lymph nodes. Three separate treatment plans, one for each phase and the corresponding PTVs have been generated with a conventional fractionation scheme of 2 Gy/fraction. For each case, a total plan (plan sum) from the sum of all phases was also created to estimate the contribution of each plan to the total dose distribution. Thus, the dosimetric parameters regarding the PTVs were compared for each phase separately, while for PTV3 the dosimetric parameters for the total plan were also compared (PTV3‐sum).

**TABLE 1:** The dosimetric parameters studied for PTV and organs at risk (OARs) per cancer type.

Structure	Dosimetric parameter examined
PTV	D_min_, D_max_, D_mean_, V_95%,_ CI
H&N OARs	
Brainstem	D_max_
Spinal cord	D_max_
Right Parotid	D_mean_
Left Parotid	D_mean_
Esophagus	D_mean_, V_45Gy_
Right optic nerve	D_max_
Left optic nerve	D_max_
Mandible	D_max_
Oral cavity	D_mean_
Lung OARs	
Lungs (volume of both lungs minus PTV)	V_20Gy_, V_5Gy_, D_mean_
Heart	V_45Gy_, V_25Gy_, D_mean_
Esophagus	V_50Gy_, D_mean_

Abbreviations: CI, Conformity Index; D_max_, maximum dose, D_mean_, mean dose; D_min_, minimum dose; D_x%_, The dose received by the x % of the corresponding structure.; H&N, Head and neck; OARs, Organs at risk; PTV, Planning target volume; V_x%_, percentage volume receiving at least x % of the prescribed dose; V_xGy_, percentage volume receiving at least x Gy.

The volumes covered by the 95% and 50% isodose curves, as well as the Conformity Index (CI) of each plan, were calculated. The CI was calculated as the quotient of the prescription isodose volume (PIV) and the corresponding PTV volume (V_PTV_) (Equation 3). It is a metric indicating the conformity of isodose curves to the size and shape of the target volume.

(3) $$\mathrm{CI}=\mathrm{PIV}/V_{\mathrm{PTV}}$$

The sum of differences between the two algorithms for each dosimetric parameter for all patients was calculated for each anatomical region separately (Equation 4).

(4) $$\Delta_{avg}\left(\%\right)=\sum \left(\frac{AXB-AAA}{AAA}\right)\;\times 100$$

where AXB and AAA represent the corresponding values of the dosimetric parameter in the AXB and AAA plans for the same patient.

Additionally, the calculation time of each treatment plan for each site with the two algorithms was compared to determine if there was a significant difference.

The paired t‐test of the IBM SPSS Statistics software (version 23) was used to assess the statistical significance of the differences with a significant level of *p* < 0.05.

#### 2.2.3 Calculation time and grid size evaluation

The dose calculation time between both algorithms, with a grid size of 2.5 mm, was compared. Additionally, in order to evaluate the dosimetric impact of the dose calculation grid size, a 1 mm grid size was considered for ten patient plans calculated with the AXB algorithm instead of the 2.5 mm, which is commonly used in treatment plans, while keeping all other parameters unchanged. These plans were then compared to the ones calculated with a 2.5 mm grid size in terms of dosimetric characteristics as well as calculation time.

#### 2.2.4 Patient plans quality assurance

In order to evaluate the effect of the two algorithms on the treatment plans overall quality the ArcCHECK (Sun Nuclear Corporation, FL) phantom was used. Patient‐Specific Quality Assurance (PSQA) for 10 patients, 5 for each anatomical site, was performed. Gamma passing rate (GP%) was used to evaluate and compare plans calculated with the two algorithms. Gamma analysis was performed with a 3% dose difference (DD) and 3 mm distance‐to‐agreement (DTA) criteria (3%/3 mm) using a 10% threshold.

## 3 RESULTS

### 3.1 Phantom study

**FIGURE 3:**
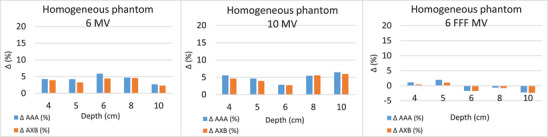
The percentage differences between the calculated (AXB and AAA) and measured by films doses for the homogeneous phantom at all measuring depths, for (a) 6 MV, (b) 10 MV, and (c) 6 MV FFF.

Comparing the calculated dose from the two algorithms with the EBT film measured dose for the case of the homogeneous phantom, AXB calculated dose showed better agreement than AAA with the dose obtained with film for all photon energies. The maximum dose differences between AXB and film were 4.5% for 6 MV, 6.0% for 10 MV, and 2.3% for 6 MV FFF, while the corresponding differences for the AAA algorithm were 5.9%, 6.5%, and −2.2%. As can also be seen in Figure 3, the AAA algorithm calculated higher doses compared to AXB, with the largest difference between algorithms being 1.35%. For energies of 6 MV and 10 MV, both algorithms calculated doses higher than the film doses, while for 6 MV FFF, lower doses were recorded.

**FIGURE 4:**
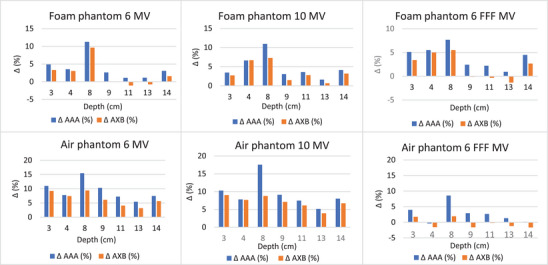
The percentage differences between the calculated (AXB and AAA) and measured doses (films) for the two heterogeneous phantoms (foam and air heterogeneities), for 6 MV, 10 MV, and 6 MV FFF at all measuring depths.

For the two heterogeneous phantoms with foam and air heterogeneity, the percentage differences between the calculated (AXB and AAA) and measured doses for 6, 10, and 6 MV FFF are shown in Figure 4. It should be noted that the depth of 8 cm, where the largest differences are observed, is the depth of the foam‐PMMA or air‐PMMA interfaces.

For the phantom with foam heterogeneity (density considered to be equal to low‐density lung tissue), the differences between calculated and measured dose values were much higher in the foam‐PMMA interface. The AXB doses showed better agreement with the measured dose. Specifically, at the interface depth of 8 cm, the dose differences between the AXB and film were 6.7 cGy (9.3%) for 6 MV, 5.4 cGy (7.3%) for 10 MV, and 3.8 cGy (5.5%) for 6 MV FFF. The corresponding differences for the AAA algorithm were 7.8 cGy (11.3%), 8.1 cGy (10.9%), and 5.3 cGy (7.7%). Also, at this depth, the calculated dose differences between the two algorithms were the highest, with AXB predicting lower doses than AAA.

For the phantom with air heterogeneity, the differences between calculated and measured doses in the air‐PMMA interface were even greater, while the calculated dose differences between the two algorithms were also higher. For 6 MV, the dose difference in the interface reached 10.3 cGy (15.5%) for AAA and 6.3 cGy (9.4%) for AXB. For 10 MV, the corresponding differences were 12.5 cGy (17.6%) for AAA and 6.3 cGy (8.8%) for AXB, while for 6 MV FFF were 5.7 cGy (8.6%) for AAA and 1.3 cGy (2%) for AXB (Figure 4). As a result, AXB showed better agreement with the measured doses since the calculated doses were lower from AAA by 5%, 7.5%, and 6% for 6 MV, 10 MV, and 6 FFF MV respectively, in air‐PMMA interface. Beyond the heterogeneity, the dose differences were smaller, with AXB also being closer to the measured doses. The films’ dose response was dependent on several factors, such as the scanner response, the time frame scanned, the field size, and the channel used, which could introduce uncertainty in the measured dose.

**FIGURE 5:**
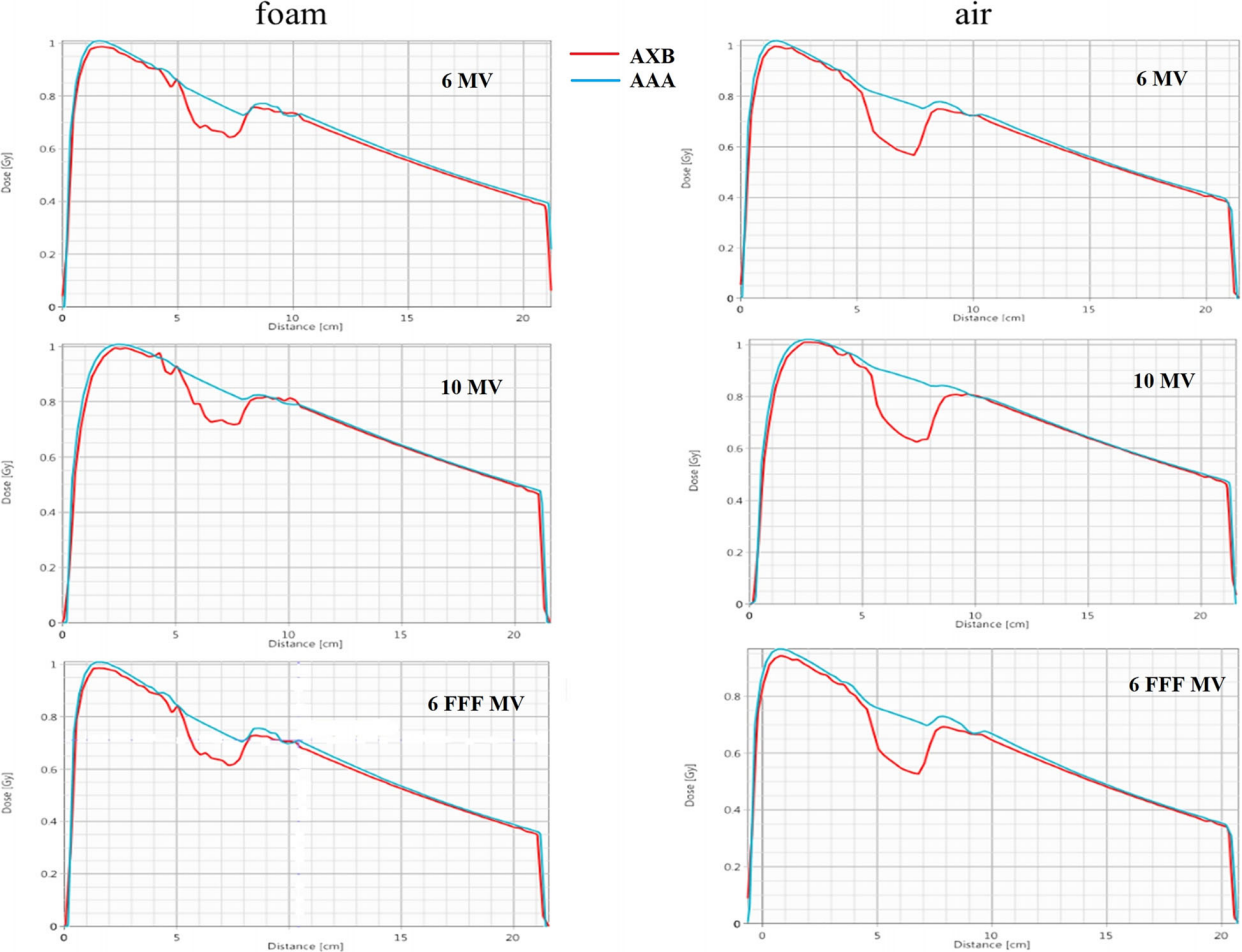
Τhe dose profiles at the central axis of the phantoms with foam (right) and air (left) heterogeneity, calculated by TPS with the two algorithms for 6 MV, 10 MV and 6 FFF MV.

Figure 5 shows the dose profiles along the central axis of the heterogeneous phantoms calculated by TPS with the two algorithms for 6 MV, 10 MV, and 6 MV FFF. It can be seen that in the low‐density region (foam or air), for example, depth region of 5–8 cm, as well as in the foam/air‐PMMA interface (at 8 cm), AAA calculated a higher dose compared to AXB for all energies. In the homogeneous regions, above and below the heterogeneities, the two algorithms calculated similar doses.

### 3.2 Clinical study

#### 3.2.1 Lung cases

For the lung cases, the relative differences between AXB and AAA algorithms for the PTV dosimetric parameters of V_95%_, D_min_, D_max_, D_mean_, and CI are given in Table 2. V_95%_ calculated by AXB was lower by an average of 6.7% compared to the corresponding AAA value. PTV D_min_ calculated by AXB was also lower by an average of 7.2%. PTV D_max_ though, was higher with AXB, while D_mean_ was lower, but not statistically significant. In comparison to AAA, AXB predicted a lower CI value (0.63 vs. 0.71). Moreover, volumes covered by 50% (V_IS50_) and 95% (V_IS95_) isodose curves were smaller when calculated by AXB.

**TABLE 2:** Relative differences between AXB and AAA algorithms and mean values concerning PTV, 50% and 95% isodose curves and OARs for lung cases.

Lung cases				
	Relative difference (AXB‐AAA)/AAA×100% Mean (Max)	AXB Mean	AAA Mean	*p*‐value
PTV				
V_95%_	−6.7 (−66.07)	87.5%	94.1%	0.02
D_min_	−7.18 (−38.37)	32.9 Gy	35.4 Gy	0.010
D_max_	1.47 (2.72)	58.3 Gy	57.4 Gy	<0.001
D_mean_	−1.21 (−13.98)	52.3 Gy	52.5 Gy	0.156
CI	−4.33 (−58.33)	0.63	0.71	0.036
V_IS50_	−1.14 (−7.74)	2200.56 cm^3^	2216.29 cm^3^	0.03
V_IS95_	−8.22 (−66.41)	674.83 cm^3^	712.43 cm^3^	0.016
Lungs (lungs minus PTV)				
D_mean_	0.45 (1.70)	14.76 Gy	14.69 Gy	0.048
V_20Gy_	0.22 (−11.8)	27.69%	27.37%	0.009
V_5Gy_	−0.08 (−3.74)	64.19%	64.41%	0.455
Heart				
D_mean_	−1.35 (−4.64)	11.84 Gy	11.98 Gy	<0.001
V_45Gy_	−0.61 (−5.12)	2.38%	2.46%	0.003
V_25Gy_	−2.39 (−5.55)	16.20%	16.62%	<0.001
Esophagus				
D_mean_	−1.67 (−3.89)	21.27 Gy	21.57 Gy	<0.001
V_50Gy_	−2.93 (−16.69)	15.93%	16.32%	0.004

Abbreviations: CI, Conformity Index; D_max_,Maximum dose; D_mean_, Mean dose; D_min_, Minimum dose; PTV, Planning target volume; V_95%_, Percentage volume receiving at least 95 % of the prescribed dose; V_ISx_, The volume covered by the x % isodose curve.; V_xGy_, percentage volume receiving at least x Gy.

**FIGURE 6:**
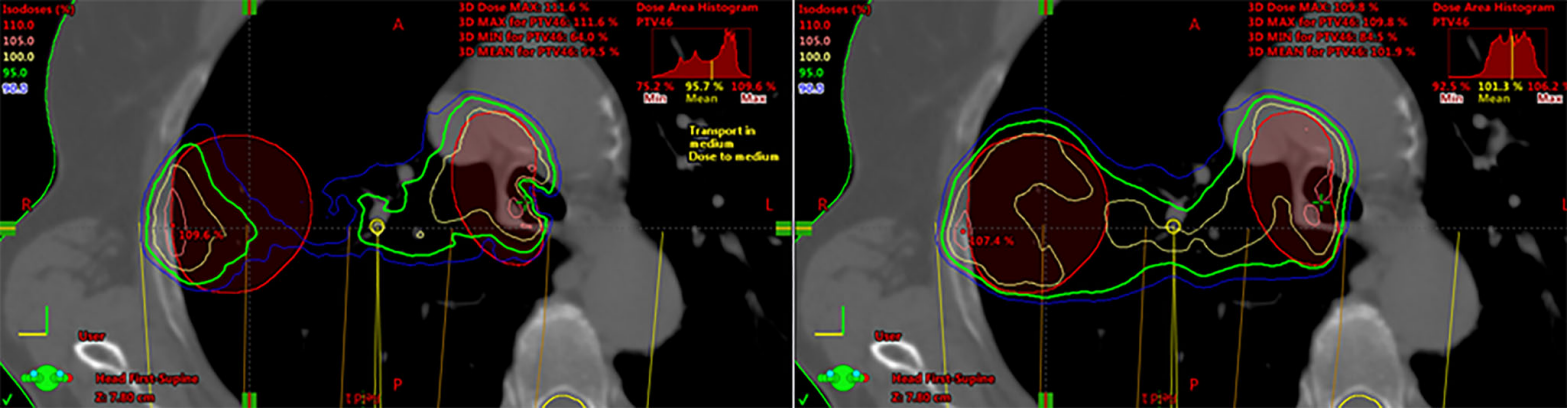
PTV (red volume) coverage for 95% isodose curve (green line) calculated by AXB (left) and AAA (right) for a single lung cancer patient.

Figure 6 shows the difference in PTV (red area) coverage for the 95% isodose curve (green line) when calculated by AXB and AAA for a single lung patient.

Table 2 also shows the relative differences between AXB and AAA algorithms for the most important dosimetric parameters of lung OARs. V_20Gy_ and mean dose to the total lung (lungs minus PTV) were calculated slightly higher with AXB compared to AAA. For the heart, AXB was found to predict lower V_45Gy,_ V_25Gy_, and mean dose values compared to AAA. For the esophagus, AXB also predicted lower V_50Gy_ and mean dose values.

#### 3.2.2 H&N cases

**FIGURE 7:**
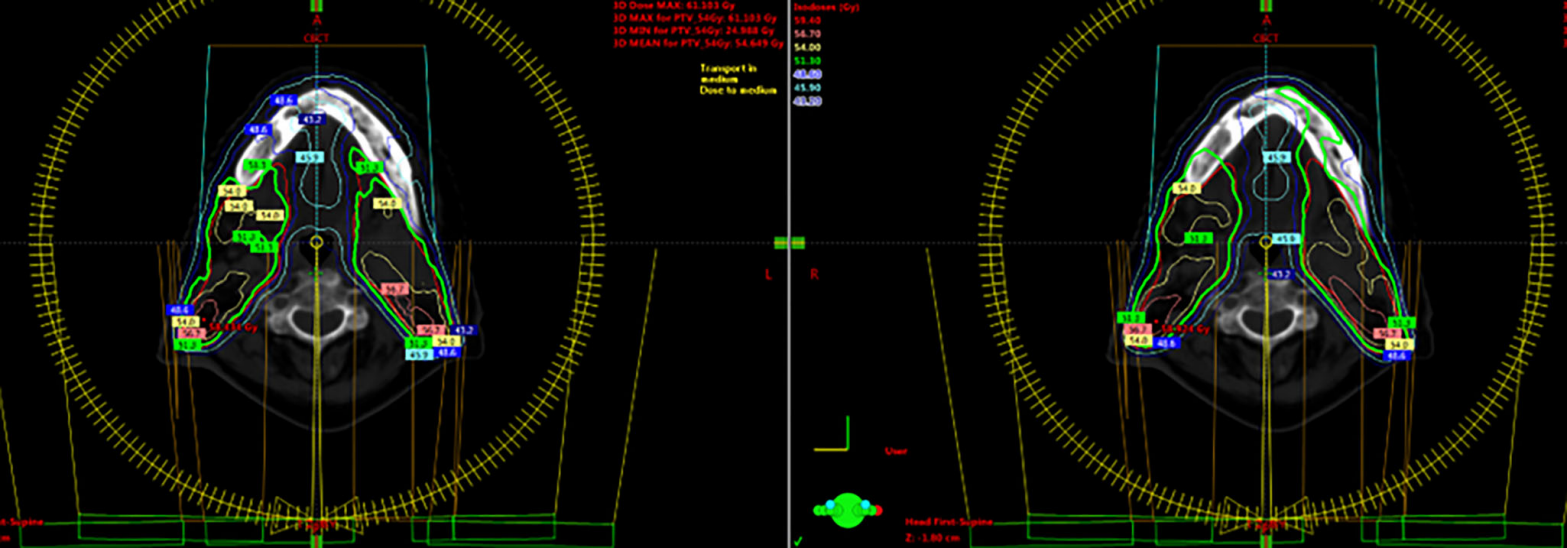
PTV coverage of 95% isodose curve (green line) calculated by AXB (left) and AAA (right) for a single H&N cancer patient.

For H&N cases, the relative differences between the two algorithms for the PTV dosimetric parameters of V_95%_, D_min_, D_max_, D_mean_, and CI are shown in Table 3. AXB predicted the V_95%_ lower by an average of 1.3% for the total plan (plan sum), while for the plans of the first, second, and third phase separately, the corresponding differences were 1.4%, 1.97%, and 2.23% respectively. For the total plan, the D_mean_ was predicted lower by AXB, compared to AAA, by 0.75% (0.7 Gy). The D_min_ was predicted lower by AXB (1.4%), while the D_max_ was higher (0.71%), but without being statistically significant. CI was found to be lower with AXB by 9.2%, 9.81%, and 8.73% for PTV1, PTV2, and PTV3, respectively. It was also found that volumes covered by 50% and 95% isodose curves were smaller when calculated by AXB (1.2% and 5.4% respectively). Figure 7 shows the coverage of the 95% isodose curve (green line) calculated by AXB and AAA for a single H&N patient. As far as the OARs are concerned, statistically significant differences were observed for the spinal cord, parotids, esophagus, left optic nerve, mandible, and oral cavity (Table 3). For all OARs, AAA overestimated the dose, D_max_ or D_mean_, compared to AXB. Specifically, in comparison to AXB, AAA calculated statistically significant higher D_max_ to the spinal cord, left optic nerve and mandible and statistically significant higher D_mean_ to the right and left parotid, esophagus, and oral cavity. The fact that a significant difference was observed in D_max_ for the left optic nerve only can be attributed to the PTV location and its proximity to the left optic nerve in some of the randomly selected cases.

**TABLE 3:** Relative differences between AXB and AAA algorithms and mean values concerning PTV, 50% and 95% isodoses and OARs for H&N cases.

Η&Ν cases				
	Relative difference (AXB‐AAA)/AAA×100% Mean (max)	AXB Mean	AAA Mean	*p*‐value
PTV3‐sum (plan sum)				
V_95%_	−1.28 (−4.18)	96.0%	97.3%	<0.001
D_min_	−1.37 (−11.6)	41.4 Gy	42.1 Gy	0.246
D_max_	0.71 (3.22)	72.6 Gy	72.2 Gy	0.093
D_mean_	−0.75 (−1.3)	65.8 Gy	66.3 Gy	<0.001
V_IS50_	−1.17 (−2.2)	1595.97 cm^3^	1577.23 cm^3^	<0.001
V_IS95_	−5.44 (−10.38)	300.4 cm^3^	317.2 cm^3^	<0.001
PTV1 (phase 1)				
V_95%_	−1.44 (−4.31)	93.8%	95.2%	<0.001
CI	−9.2 (38.1)	0.68	0.76	0.01
PTV2 (phase 2)				
V_95%_	−1.97 (−5.16)	93.4%	95.3%	<0.001
CI	−9.81 (−18.03)	0.74	0.82	<0.001
PTV3 (phase 3)				
V_95%_	−2.23 (−5.59)	93.7%	95.8%	0.001
CI	−8.73 (−23.88)	0.75	0.81	<0.001
Brainstem				
D_max_	−1.54 (−5.78)	30.23 Gy	30.61 Gy	0.452
Spinal Cord				
D_max_	−2.15 (−3.47)	33.88 Gy	34.62 Gy	<0.001
Right Parotid				
D_mean_	−1.65 (−3.46)	24.64 Gy	25.02 Gy	<0.001
Left Parotid				
D_mean_	−1.72 (−3.46)	22.13 Gy	22.52 Gy	<0.001
Right optic nerve				
D_max_	−0.15 (−1.71)	34.06 Gy	34.16 Gy	0.448
Left optic nerve				
D_max_	−1.08 (−2.18)	27.97 Gy	28.48 Gy	0.021
Esophagus				
D_mean_	−1.69 (−4.69)	20.85 Gy	21.16 Gy	<0.001
V_45Gy_	−2.18 (−14.25)	20.53%	20.85%	0.078
Mandible				
D_max_	−1.83 (−3.02)	63.72 Gy	64.90 Gy	<0.001
Oral cavity				
D_mean_	−0.8 (−1.7)	39.43 Gy	39.74 Gy	<0.001

Abbreviations: CI, Conformity Index; D_max_, Maximum dose; D_mean_, Mean dose; D_min_, Minimum dose; PTV, Planning target volume; V_95%_, Percentage volume receiving at least 95 % of the prescribed dose; V_ISx_, The volume covered by the x % isodose curve.; V_xGy_, percentage volume receiving at least x Gy.

Furthermore, a clinical study of 20 prostate cases was conducted. However, the differences found were not significant, and the results are not reported in the present study. It was concluded that the two algorithms calculated similar doses in anatomical sites with small heterogeneities, such as the pelvis.

#### 3.2.3 Calculation time and grid size evaluation

Non‐significant differences were found in dose calculation times between algorithms using a grid size of 2.5 mm for all plans. Calculation times were similar, with AXB being either faster or slower than AAA depending on the complexity of the clinical plan. Using a grid size of 1 mm instead of 2.5 mm for dose calculations with AXB, it was found that the difference in dosimetric impact was negligible for all dosimetric parameters, but the calculation time increased dramatically. Specifically, while the average calculation time with a grid size of 2.5 mm was 2.3 min, this time reached 22.8 min with a grid size of 1 mm. In some cases, the calculation time became even more than 10 times longer.

#### 3.2.4 Patient plans quality assurance

The average GP% of the QA plans calculated with AXB and AAA, for each anatomical site separately and for the total plans, is shown in Table 4. Gamma analysis evaluation showed that the GP% of each plan calculated with both algorithms was higher than 95%. The comparison of GP% for the total plans showed a statistically significant difference, with the AAA appearing to exhibit dose distributions with slightly higher GP%. However, this difference did not negatively affect the AXB QA results since the plans calculated with AXB had a high GP%, equal to 99% on average. Regarding the comparison of the QA plans per anatomical region, a statistically significant but still negligible difference appeared to be only for H&N plans, with the AAA plans having a higher GP%.

**TABLE 4:** Gamma passing rates (GP) comparison of plans calculated with both algorithms at 3% dose distance (DD) and 3 mm distance to agreement (DTA) criteria.

	GP_ΑΧΒ_ (%)	GP_ΑΑΑ_ (%)	GP_AXB_‐GP_AAA_ (%)	*p*‐value
H&N	98.3	99.1	−0.72	0.026
Lung	98.8	99.5	−0.68	0.132
Total	98.5	99.2	−0.71	0.003

Abbreviations: GP, gamma passing rate; H&N, head and neck.

## 4 DISCUSSION

### 4.1 Phantom study

The phantom study showed that doses calculated by AXB were in better agreement with the corresponding doses measured by the films, especially at the interface regions, where AAA calculated higher doses. Several studies involving heterogeneous phantoms have shown that doses calculated by AXB were closer to Monte Carlo simulations than those of AAA, concluding that AXB appears to be more accurate in cases of heterogeneity.^20^, ^21^, ^22^ A study by Bush et al. with a phantom containing an air cavity irradiated with a 6 MV photon beam, showed that in the secondary build‐up region beyond air, the difference between AXB and MC was up to 4.5%, which increased to 13% with AAA, underestimating the secondary dose buildup.^21^ The two algorithms were compared at interfaces of different materials, and it was found that AXB calculated significantly lower doses than AAA at the air‐tissue interface, which is in agreement with the results of the current study. Likewise, Kroon et al. compared the doses measured by EBT2 films with those calculated by the algorithms AAA and AΧΒ in a PMMA phantom containing an 8 cm foam layer with low density (ρ = 0.03 g/cm^3^) and found that the relative doses were much higher for AAA in the low‐density layer.^13^

In accordance with the above studies, the present study showed that AAA overestimated the dose distribution, especially in the air or low‐density cavities and in air‐tissue interfaces beyond the air. This outcome can be attributed to the modeling of scattered radiation contribution, which sets a limitation in dose calculation accuracy in secondary build‐up regions beyond low‐density heterogeneities. Based on these results, a dose overestimation in treatment plans calculated by AAA is expected, especially in low‐density heterogeneity regions. Therefore, caution is required for a PTV underdosage not to occur.

### 4.2 Clinical study

The dosimetric analysis of the lung treatment plans showed that V_95%_ was notably lower when calculated with AXB. In some cases, this difference was negligible, while in others it exceeded 5%, with the V_95%_ value being significantly lower than 95% with AXB. A few extreme cases with higher V_95%_ differences reaching up to 17.9% and 66% were also observed, which indicate a significant underdosage of PTV when the treatment plan is calculated with AAA. These high differences could be attributed to the target position within the lung, as well as to the low‐density volume included in the PTV. Specifically, they refer to cases where the PTV is small, located in the central area of the lung lobe, and contains a large volume of low‐density tissue (e.g., air). For example, in the case presented in Figure 6, the V_95%_ appears to be equal to 99.6% with AAA, whereas it is equal to 81.8% with AXB. These results could be explained by taking into account the properties of each algorithm, for example, the improved accuracy of the AXB algorithm compared to AAA in the absence of electron equilibrium conditions, where the latter overestimates the dose.^23^

The aforementioned results are consistent with those of Kroon et al., who assessed the dosimetric impact of AAA and AXB for lung plans and observed a serious underdosage of PTV up to 12.3% when treatment plans of stage I lung patients were calculated with AAA.^13^ For stage III patients, the difference in PTV coverage was smaller due to larger fields and tumor sizes, resulting in smaller errors in the AAA algorithm. Similar results have also been stated in other studies.^23^, ^24^

Furthermore, in this study, the lung dose calculated by AAA was in some cases higher, while in other cases lower than AXB, in accordance to Bush et al. who reported that AAA can either overestimate or underestimate the lungs dose, depending on field size, target location, and lung density.^21^ The dosimetric parameters of V_20Gy_ and mean dose were calculated slightly higher with AXB, which indicates that treatment plans calculated with AAA could slightly underestimate the lungs' V_20Gy_ and mean dose values. Similar results were found by Kroon et al. regarding lung dose.^13^ They also found small differences for the other OARs doses without giving any further details in their study. Another study by Fleming et al. analyzed the impact of AXB on VMAT plans of patients with locally advanced non‐small cell lung cancer (LA‐NSCLC) and similarly concluded that AAA predicted statistically significant higher doses to spinal cord and esophagus, whereas they did not find a statistically significant difference for mean dose, V_20Gy,_ and V_5Gy_ of lungs.^24^

The analysis of H&N plans showed that treatment plans calculated with AAA may lead to a PTV underdosage, which depends on the target location and size. There are also other factors that can affect the PTV dose coverage, namely the PTV‐to‐skin distance^25^ and photon energy.^26^ The highest relative differences (>3%) of PTV V_95%_ were observed in cases where PTV included air cavities or bones. Similarly, to the lung cases, the differences in PTV coverage are based on the fact that AAA predicts higher doses in secondary build‐up areas beyond air cavities or bones, where there is no electronic equilibrium. In the H&N case shown in Figure 7, the reduced coverage of the 95% isodose curve for the AXB calculated plan, is evident in the mandible area. This results in reduced PTV coverage, which is adjacent to the mandible, with V_95%_ calculated equal to 93.7% with AXB instead of 96.5% calculated with AAA.

Furthermore, AAA overestimated the dose of all OARs studied for the majority of the H&N cases. The highest absolute difference was observed for the mandible D_max_, which was calculated 1.2 Gy lower with AXB as expected, since the mandible included bone with much greater Z than soft tissues.

The above results are consistent with the Carles Muñoz‐Montplet et al. study.^17^ They compared AAA and AXB algorithms for 110 H&N patients treated with VMAT and concluded that AAA overestimates all dosimetric parameters concerning PTV and OARs (0.2 Gy‐2.4 Gy), with the largest differences detected for the D_max_ to the cochlea and the mandible. Similar results have also been found in other studies, highlighting the fact that the reduced doses calculated by AXB are mainly due to the presence of bone or air in the OARs.^27^, ^28^, ^29^

Another outcome of this study was the fact that CI was significantly lower with AXB compared to AAA for the treatment plans of all anatomical sites studied. This lower conformity could be explained by considering the fact that only the final dose calculation was performed with different algorithms, whereas the optimization result and hence fluence generation were performed with AAA as a standard choice. The optimization objectives were kept unchanged in order to study the effect of the AXB only on the final dose calculation step. Likewise, the volumes covered by the 50% and 95% isodose curves were calculated smaller with AXB. These outcomes indicate the possible PTV underdosage with the AAA algorithm and not the AXB plans inferiority.

The calculation time was not found to differ significantly between the two algorithms (*p* < 0.05) for dose calculation grid size 2.5 mm. When changing the grid size from 2.5 to 1 mm for dose calculation with AXB only, calculation times increased notably, but the dosimetric impact was negligible. This fact should be taken into account when adopting the 1 mm grid in the clinical routine. Nevertheless, PTV size, hence the field size, can affect both the dosimetric result and the calculation time. The same result was published by Kroon et al. and Kim KH et al., who compared the impact of the two different grid sizes for AXB, in terms of calculation time and plan quality in lung and prostate plans, respectively.^13^, ^15^

Finally, the choice of the algorithm did not seem to affect the treatment plan quality in terms of GP%. This result is not in agreement with other studies that have shown that the GP% of VMAT plans calculated with AXB was higher for various acceptance criteria, including the 3%/3 mm used in this study.^30^, ^31^ A further investigation on this matter could include the analysis of the measured plans with acceptance criteria other than the ones adopted in this study, such as the criteria of 3%/2 mm, 2%/3 mm, etc.

From our study of prostate plans, we conclude that AXB is best for sites with large heterogeneities, not typically an issue in the pelvis.

This study aimed to provide the reader with a complete and thorough view of the dosimetric characteristics of the AXB algorithm and how it compares to the AAA. We regard this work to be a continuation of the aforementioned works, and at the same time, to the best of our knowledge, we provide a phantom‐based and clinical‐based correlation of the behavior of AXB (with respect to AAA) that appears to be lacking in the literature. We have tested and compared the algorithms in several dosimetric parameters regarding PTVs and OARs, treatment sites, PSQA, as well as grid size, etc. This thorough analysis was performed in order to inspect how each aspect of treatment planning can be affected by the use of AXB. Our results are in good agreement with previously published works, and at the same time, they provide a deeper insight into the aspects of using AXB in clinical practice.

With regards to the limitations of the study, a third arm consisting of comparing plans which would have been both optimized and calculated using each of the two algorithms could provide a wider overview of the overall clinical impact of the performance of both algorithms in the treatment planning procedure. In addition, a further comparison of the calculated dose with a measured one using an ionization chamber, despite its inherent difficulties (dose at material interface), could provide insight into the observed discrepancies between films and algorithms. Finally, a comparison between AAA and AXB using 1 mm calculation grid could help in investigating its impact on overall treatment plan quality (dose distribution, deliverability, gamma passing rate, etc.).

## 5 CONCLUSIONS

The results from our phantom study showed that the AXB algorithm more accurately calculates the dose distribution compared to AAA, as the latter overestimates the dose, especially in air‐tissue interfaces beyond air cavities, due to the modeling of scattered dose. Subsequently, similar results were obtained from the comparison of the lung and H&N treatment plans. It was observed that AAA often overestimates the PTV coverage, specifically when the target contains air cavities or low‐density tissues. This fact could lead to a PTV underdosage, which in turn might lead to a possible relapse of the disease. Furthermore, the calculation time was not found to differ significantly between the two algorithms using a grid size of 2.5 mm. However, the use of 1 mm grid size for dose calculation with AXB resulted in a notable increase in calculation time without inducing a significant difference in plan quality. Finally, the algorithm choice did not seem to affect the result of patient QA in terms of GP%. In conclusion, the choice of the AXB algorithm over the AAA algorithm for H&N and lung VMAT treatment plans seems to offer substantial advantages including more accurate dose calculation, similar calculation times, and overall high plan quality.

## AUTHOR CONTRIBUTION

Conceptualization, Kalliopi Platoni; Methodology, Kalliopi Platoni, George Patatoukas, Maria Tsimpoukelli; Investigation, Maria Tsimpoukelli, George Patatoukas, Nikolaos Kollaros, Vassilis Kouloulias, Andromachi Kougioumtzopoulou, Dimitra Michaletou; Writing—original draft preparation, Maria Tsimpoukelli, Marina Chalkia; Writing—review and editing, Maria Tsimpoukelli, Marina Chalkia, Kalliopi Platoni; Supervision, Kalliopi Platoni All authors have read and agreed to the published version of the manuscript.

## CONFLICT OF INTEREST STATEMENT

The authors declare no conflicts of interest.
